# Acoustic asymmetric transmission based on time-dependent dynamical scattering

**DOI:** 10.1038/srep10880

**Published:** 2015-06-03

**Authors:** Qing Wang, Yang Yang, Xu Ni, Ye-Long Xu, Xiao-Chen Sun, Ze-Guo Chen, Liang Feng, Xiao-ping Liu, Ming-Hui Lu, Yan-Feng Chen

**Affiliations:** 1 National Laboratory of Solid State Microstructures & Department of Materials Science and Engineering, Nanjing University, Nanjing 210093, China; 2 Department of Electrical Engineering, The State University of New York at Buffalo, Buffalo, NY 14260, USA

## Abstract

An acoustic asymmetric transmission device exhibiting unidirectional transmission property for acoustic waves is extremely desirable in many practical scenarios. Such a unique property may be realized in various configurations utilizing acoustic Zeeman effects in moving media as well as frequency-conversion in passive nonlinear acoustic systems and in active acoustic systems. Here we demonstrate a new acoustic frequency conversion process in a time-varying system, consisting of a rotating blade and the surrounding air. The scattered acoustic waves from this time-varying system experience frequency shifts, which are linearly dependent on the blade’s rotating frequency. Such scattering mechanism can be well described theoretically by an acoustic linear time-varying perturbation theory. Combining such time-varying scattering effects with highly efficient acoustic filtering, we successfully develop a tunable acoustic unidirectional device with 20 dB power transmission contrast ratio between two counter propagation directions at audible frequencies.

The phenomenon of unidirectional motion for matters or particles in a gradient potential has inspired physicists for many centuries and fruitful discoveries by these physicists have led to multiple extraordinary inventions including hydroelectric power and electric diodes, which have undoubtedly revolutionized our society. Recently, the research of such important phenomenon has been extended to include even more particles and quasi-particles, e.g., photons represented by optical waves^1^,^2^,^3^, phonons represented by acoustic or elastic waves^4^ and thermal phonons represented by thermal waves^5^,^6^. For photons, several physical phenomena can be exploited to break the reciprocity to allow for unidirectional transmission and even optical isolation, e.g., non-reciprocal Faraday effect in a linear system^7^,^8^ and direction-dependent frequency conversion in a time-varying modulation system^3^,^9^. Similarly, for phonons or acoustic waves, acoustic diodes^10^ exhibiting unidirectional acoustic wave transmission^11^,^12^,^13^,^14^,^15^,^16^,^17^,^18^,^19^ may be realized in several configurations. For example, acoustic diodes or circulators have been realized in linear systems with magneto-acoustic coupling effects^20^ or with acoustic analogue of Zeeman effects in moving media^4^. Besides, acoustic rectifiers and diodes have also been demonstrated using frequency conversion processes in passive acoustic nonlinear systems^21^,^22^ and a hybrid acoustic/electric active system with nonlinear functionality implemented in the electric domain^23^,^24^. However, practical implementations of these systems have several drawbacks including the need of a high input energy, or limited tunability and operation bandwidth. In this letter, we present a new acoustic frequency conversion mechanism through the acoustic wave’s passive interaction with a rotating object, *i.e*., an elliptical blade with its surrounding air. Starting with an acoustic linear time-varying perturbation theory, we develop a method to solve the problem of acoustic wave scattering through a time-varying medium, by which we can fully describe the underlying mechanism responsible for the frequency conversion. Our theoretical results show an excellent agreement with simulation results obtained from a time-varying finite element method. By combining such time-varying medium with a deliberately designed acoustic filtering structure in an acoustic guiding system, we develop an acoustic asymmetric device with unidirectional propagation exhibiting 20 dB contrast ratio between two propagation directions at audible frequencies.

## Results

### Time-varying medium scattering effect

**Figure 1:**
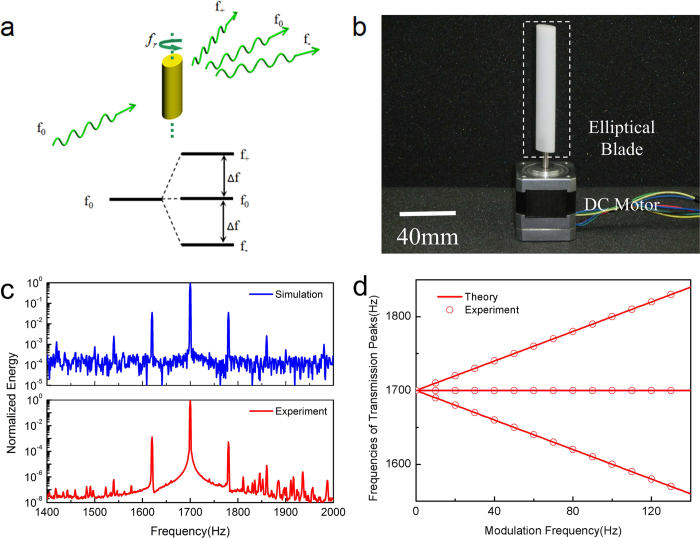
Time-varying medium scattering effect. (**a**) Illustration of time-varying acoustic scattering from an acoustic time-varying medium, represented by an elliptical-shaped blade rotating around its center axis in the air. Acoustic time-varying scattering interaction produces extra harmonic frequency components (*f*_−_ and *f*_+_). (**b**) Photography of the time-varying medium which is represented by a nylon elliptical-shaped blade driven by a precisely controlled DC motor. (**c**) The top plot shows time-varying full-wave acoustic transmission simulation results (blue solid line) for an input acoustic wave with a frequency *f*_0_ = 1700 *Hz* transmitting through a rotating elliptical blade (8 mm long semi-major axis and 4 mm long semi-minor axis) centered in an acoustic waveguide with a width of 22.5 mm with its rotating frequency *f*_*r*_ = 40 *Hz*. Note that the rotating motion of the blade is modeled as a sinusoidal time-varying density parameter in the simulation (see Fig. S.1 in Supplementary Information for details). The 1620 Hz and 1780 Hz frequency components observed in the transmission spectrum correspond to the scattering products. There are also secondary cascaded scattering products peaked at 1540 Hz and 1860 Hz. The bottom plot shows the experimental transmission spectrum (red solid line) obtained using a fabricated time-varying medium with the same geometric parameters as in the simulation. Without taking into account the higher order cascaded scattering terms, the experimental spectrum agrees well with the numerical simulated result. Note the harmonic scattering components on the high frequency side of the spectrum, which might originate from the nonlinear effect of the forced motion of the blade’s surrounding air. (**d**) The frequency of the primary high order scattering components as a function of blade’s rotation frequency *f*_*r*_. Circles correspond to experimental data while lines correspond to the results obtained from the theory outlined in Supplementary Information.

The interaction of waves and static matter has been widely explored since the early days of physics. In recent years, the interaction of waves with non-stationary medium^25^, whose mass density and wave velocity are assumed to be time-varying, has raised considerable interests. In this letter, we propose a new kind of time-varying medium, composed of a rotating elliptical blade with its surrounding air, of which the schematic is shown in Fig. 1a and the photograph is shown in Fig. 1b. According to the full wave simulation, the effective mass density of the rotating blade are dependent on the rotating angle, which is defined as an angle between the propagation direction of plane waves and the majoraxis of elliptical blade (shown in Fig. S.1. in Supplementary Information). Because the time-varying angle is a linear function of the blade’s rotating frequency, the material elastic parameters are time-varying and modulated correspondingly. Such periodically varying material properties of the scatters placed in an acoustic waveguide will change the propagation behavior of the input acoustic waves and result in a harmonic scattering phenomenon. Consequently, this would lead to the energy transfer from the fundamental frequency to the harmonic waves, which acquire a lower and higher frequency as indicated in Fig. 1c. Since the acoustic field of the effective time-varying region might be inhomogeneous, measuring the field in the time-varying region at several discrete locations is insufficient in figuring out the parameters of the whole time-varying region. And the limits of present measurement techniques also prevent us from directly measuring the whole sound field in the time-varying region. Furthermore, because of the uncertainty principle in the time-domain measurement, it is of huge difficulties using the current transient measurement method to deduce the time-varying parameters from the recorded transmission and reflection waves with only finite recording length of signals. Taking all these factors into consideration, we resort to a quasi-static approximation method to deduce the effective parameters of modulation region from reflected and transmitted waves. (see Theory in Supplementary Information for details).

Herein, the shape of the rotating blade is chosen to be an elliptic instead of other common shapes, because the air turbulence^26^ caused by the rotational motion is smaller for the elliptical blade than, for example, a rectangular blade. Slow varying approximation can thus still be preserved in the theoretical analysis. In this paper, we develop a method by utilizing an acoustic linear time-varying perturbation theory to study the scattering process of acoustic waves transmitting through a time-varying medium. Using such method we can fully describe the frequency conversion effect of scattering waves as follows.

Replacing velocity field 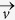 by velocity potential 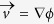, the acoustic equations can be described as:


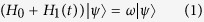


where *ω* is the eigen frequency, with *H*_0_ and *H*_1_(*t*) expressed as


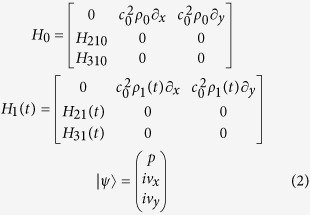


In the above analysis, we have set:


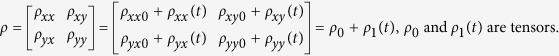


In the presence of rotation and considering the two-folded symmetry of the elliptical blade, the effective Hamiltonian *H*_1_(*t*) can be written as 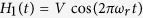 (refer to Theory in Supplementary Information for more theoretical details), where *ω*_*r*_ is the rotation angular frequency of the blade. The new eigenvectors can be written as a linear superposition of 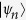 as:


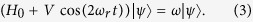


By solving the above equation (refer to Theory in Supplementary Information for more theoretical details), we find that the initial system energy level will be split to form two additional sub-energy levels as shown in Fig. 1a. The corresponding angular frequencies for these two sub-energy levels are determined according to the following expression,


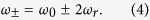


Shown in Fig. 1c (blue solid line) is the transmission spectrum of the acoustic waves propagating through a waveguide obtained from full-wave simulations conducted in COMSOL Multiphysics (see Methods). Clearly when the time-varying medium in the waveguide is brought to a time-varying state corresponding to the rotation motion of the blade, the obtained acoustic transmission spectrum shows several important features. There is still a large unconverted spectral component at the input frequency of 1700 Hz, indicating relatively low conversion efficiency for this single-pass time-varying scattering process. Nevertheless, the transmission spectrum contains newly generated scattering frequency components with two major peaks at 1620 Hz and 1780 Hz, which are converted from the input acoustic wave. Notice that the first order frequency shift is twice the rotating frequency of the blade, which is expected from the two-folded geometric symmetry of the elliptical blade. In addition, due to a secondary order scattering effect, there are also much weaker peaks at 1540 Hz and 1860 Hz.

Figure. 1b shows a picture of the fabricated time-varying medium consisting of an elliptical blade made of nylon driven by a precisely controlled brushless DC motor. The acoustic transmission property through this time-varying modulated medium is experimentally investigated (see Methods) with results shown in Fig. 1c (red solid line). The frequency shifts are clearly visible in the experiment results, which coincides with the simulated data. Both experiments and simulations indicate that time-varying modulation can in fact lead to a dramatically altered transmission properties of the fundamental frequency waves. Note that due to a noisy background in our experiments, the spectral position of the secondary cascaded scattering products cannot be unambiguously distinguished. The frequency conversion efficiency observed in the experiments is somehow lower than that in the simulations. This is primarily due to the non-ideal experimental conditions, e.g., the nonlinear effects caused by the moving air around the blade and energy loss in the waveguide. One of the advantages of using time-varying modulation lies in the fact that the frequency of scattered waves can be directly manipulated by controlling the blade’s rotation frequency (*f*_*r*_) as shown in Fig. 1d. More specifically, the frequency of scattered waves shows a linear relationship with *f*_*r*_, which is also consistent with the theoretical result shown in Eq. 4. Exploiting such dependence enables an extra design freedom for acoustic devices exhibiting unidirectional transmission characteristics.

### Unidirectional acoustic system design

**Figure 2:**
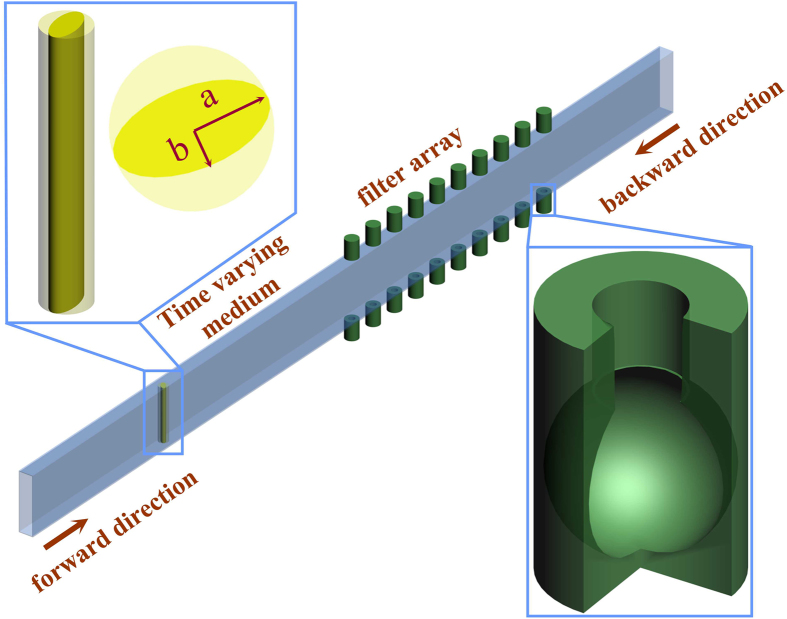
Schematic illustration of a unidirectional transmission system. It is a two-component device consisting of a time-varying medium region and an acoustic filter region, both of which are enclosed in a rectangular acoustic waveguide. A rotating elliptical-shaped blade represents the time-varying medium. The acoustic filter is a band-stop filter constructed with an array of Helmholtz resonators. Propagation directions in this paper are defined as follows: forward propagation defines the direction along which the input acoustic wave first encounters the time-varying medium and then the acoustic filter, and the opposite propagation direction is defined as backward propagation. In a forward propagation, part of the input acoustic wave is scattered into high order scattering components, and if the frequency of these scattering products are out of the filter’s stop-band, they can pass through the filter leading to certain power transmission. But in a backward propagation, if the frequency of input acoustic wave falls into the filter’s stop-band, it will be filtered out and attenuated, leading to nearly no power transmission regardless of the scattering process occurred at the blade. The power transmission contrast in these two propagation directions characterizes the unidirectional behavior of this system.

Shown in Fig. 2 is the schematic of our proposed unidirectional acoustic transmission system consisting of two major components: a time-varying medium region as described in the above section and an acoustic band-stop filter constructed with an array of cascaded Helmholtz resonators. Usually Helmholtz resonators can have high quality factors and their resonance frequencies can be accurately tailored by changing their geometry. Such unique property of Helmholtz resonators allows one to design a filter with high extinction ratio and with desired bandwidth and center frequency. Therefore the transmitted acoustic power through this two-component system for the forward (from time-varying medium to band-stop filter) and backward propagation (from band-stop filter to time-varying medium) can be expressed as follows:


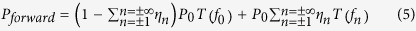



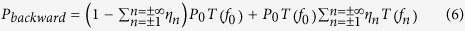


Here, *P*_0_ and *f*_0_ are power and frequency of the input acoustic wave respectively, *T*(*f*) is the frequency-dependent normalized power transmission function of the filter, *η*_*n*_ and *f*_*n*_ are the conversion efficiency (defined as the ratio between converted power and input power: *P*_*n*_/*P*_0_) and frequency of the *n*^th^ order acoustic scattering product. In the above equations, the first and second term describes the power transmission for the input acoustic wave and for all converted multiple-order scattering components respectively. Clearly the power transmitted for the input acoustic wave is exactly the same for both propagation directions. However, for the frequency band of interest, *i.e*., the filter’s stop-band, the transmitted multiple-order scattering components’ power is different due to the fact that the incident power for the time-varying acoustic scattering process at the elliptical blade highly depends on the propagation direction. In a forward propagation case, the input acoustic wave encounters the time-varying medium first, indicating that the incident power for the time-varying scattering process is approximately the same as the input power, while in a backward propagation case, the input acoustic wave is first filtered and attenuated heavily by the Helmholtz filter before it encounters the time-varying medium, in which case the incident power is much lower. The difference in the total transmitted power Δ*P* for the two directions can be expressed as:


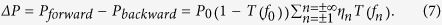


Depending on the property of the band-stop filter *T*(*f*), especially its bandwidth and center-frequency, and the high order scattering frequency *f*_*n*_ governed by the rotating frequency of the blade *f*_*r*_ as well as the input frequency *f*_0_, the value of Δ*P* can either be zero or positive, corresponding to symmetric transmission or unidirectional transmission.

**Figure 3:**
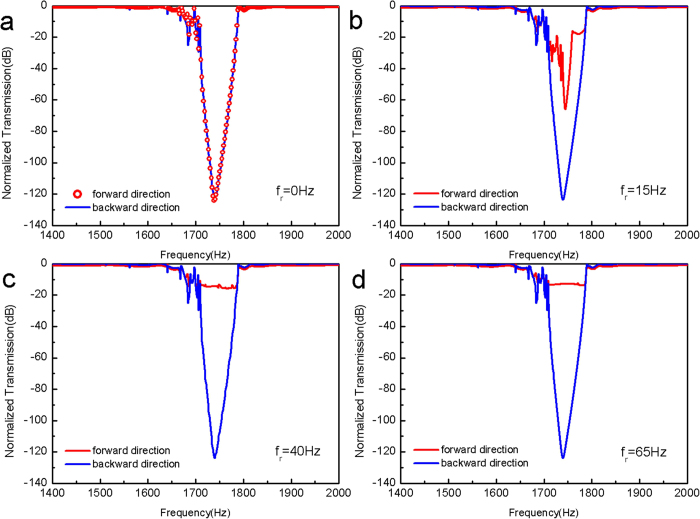
Full-wave simulation results for acoustic transmission through our designed system. (**a**) Normalized transmission spectra when blade is at rest with rotating frequency *f*_*r*_ = 0. The spectra for forward and backward propagation directions are nearly identical within the numerical error in the simulation, and they correspond to the filter’s intrinsic frequency response. In this case, the system transmission property is symmetric. (**b–d**) Normalized transmission spectra corresponding to three different excited states/rotating frequencies of the blade: (**b**) *f*_*r*_ = 15 *Hz*, (**c**) *f*_*r*_ = 40 *Hz*, (**d**) *f*_*r*_ = 65 *Hz*. With the increasing rotating frequency of the blade, the system starts to develop asymmetric response with increasing power transmission for the forward propagation direction but almost unchanged power transmission power for the backward propagation, which leads to an increased power transmission contrast ratio for these two propagation directions. At *f*_*r*_ = 15 *Hz* asymmetric transmission is observed primarily for input frequency near the center of the filter’s stop-band, while at higher frequencies *f*_*r*_ = 40 *Hz* and *f*_*r*_ = 65 *Hz* asymmetric frequency response occurs for most of input frequencies falling into the filter’s stop-band. At the highest rotation frequency of *f*_*r*_ = 65 *Hz* used in our experiment, the power transmission contrast ratio is close to 100 dB.

Our proposed unidirectional system is implemented and numerically studied in COMSOL Multiphysics (See Methods). When the elliptical blade is at rest meaning a zero rotation frequency *f*_*r*_ = 0, as shown in Fig. 3a this system functions merely as a regular filter with numerical-error limited symmetric transmission property, and the obtained power transmission spectrum reflects exactly the filter’s frequency response. This result is consistent with our above analysis and is expected from Eq. (7) because there is no scattering term and the system only has a filter response. In the case for a rotating blade, the resulted scattering process greatly modifies the transmission spectrum as illustrated in Fig. 3(b–d) corresponding to blade rotation frequencies *f*_*r*_ of 15 Hz, 40 Hz and 65 Hz, respectively. First of all, when the input frequency falls out of the stop-band of the filter (e.g. frequency > 1800 Hz), this two-component system acts as if the filter did not even exist and there is no direction-dependent power transmission, suggesting symmetric transmission spectrum for such frequencies. However, for input frequencies falling within the filter’s stop-band, the transmitted power increases with the increasing *f*_*r*_ in a forward propagation configuration, but remains almost unchanged in a backward configuration. This observation can be well explained by our theoretical framework outlined in the previous section. As indicated by Fig. 1(d), the change of *f*_*r*_ causes a linear shift for the scattering frequency components. According to our analysis and Eq. (7), increasing the rotating frequency *f*_*r*_ pushes the generated scattering frequency components to the edge of or even out of the filter’s stop-band, leading to increased transmitted power difference for the two propagation directions.

### Measurement results

**Figure 4:**
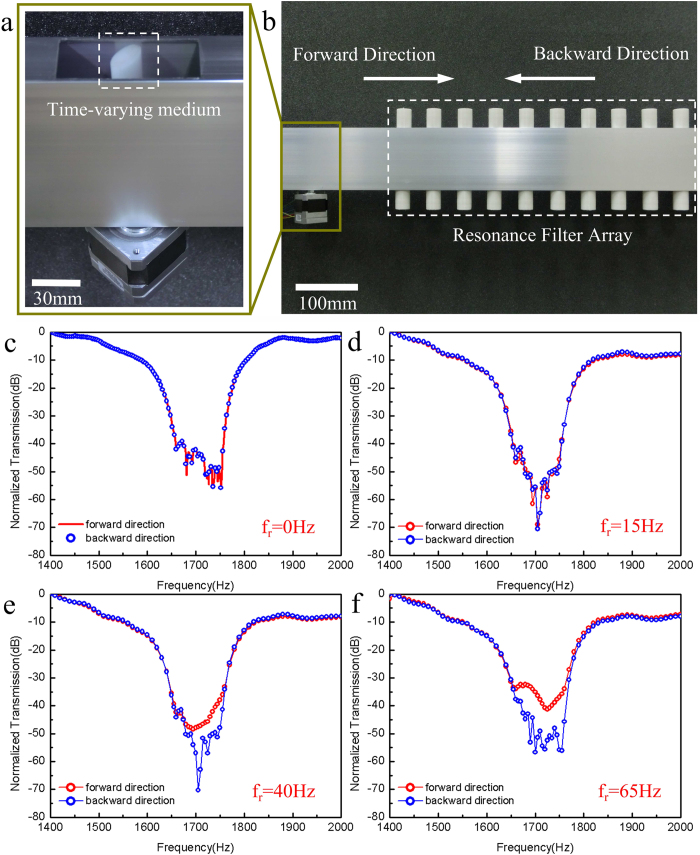
Experimental system and results. (**a**) Photography of the time-varying medium installed in an acoustic waveguide with an opening at the top. The time-varying medium is represented by a nylon elliptical-shaped blade driven by a precisely controlled DC motor. (**b**) Photography of the two-component acoustic wave-guiding system, consisting of a time-varying medium region and a Helmholtz-resonator-array-based acoustic band-stop filter. The definition of forward and backward propagation direction in the experiment is the same as that in the simulation shown in Fig. 2(c–f) Measured normalized power transmission spectra of this system for four different configurations: (**c**) without the presence of the blade in the system, (**d**, **e** & **f**) rotation frequency *f*_*r*_ = 15 *Hz*, *f*_*r*_ = 40 *Hz*, and *f*_*r*_ = 65 *Hz* respectively. Similar to our numerical investigations, this experimental system experiences a transition from symmetric transmission behavior into asymmetric behavior and consequently an increase in contrast ratio. However, compared with our numerical simulation results, this transition occurs at a higher rotating frequency, because the fabricated filter has a broader bandwidth than the numerically modeled one and a larger rotating frequency is necessary to induce a larger frequency shift so that the high order scattering frequency components can acquire frequencies out of the filter’s stop-band. At the maximum rotation frequency (65 Hz), this device shows a unidirectional transmission property with a contrast ratio up to 20 dB.

A full experimental test system is built based upon the simulated structure. Photographs of the blade unit (time-varying medium) and the whole device are presented in Fig. 4(a,b). Multiple Helmholtz resonators are made of plastic and cascaded together to form an array with a resonance frequency around 1700 Hz. More details of this system are provided in Supplementary Information. Without the physical presence of the elliptical blade in the waveguide system, as shown in Fig. 4(c) nearly symmetric normalized power for the forward and backward propagation direction transmission (corresponding to the filter response) is obtained. Notice that the bandwidth of this fabricated filter array’s stop-band is broader than that of the numerically modeled one shown in Fig. 3(a) (3-dB bandwidth: 100 Hz vs. 20 Hz). The discrepancy can be ascribed to a lower resonant Q factor for the experimentally fabricated Helmholtz cavity due to acoustic propagation loss in air (as shown in Supplementary Information Fig. S.8.) and the non-ideal rigid wall condition. When the time-varying medium is introduced into the system but is at rest (*f*_*r*_ = 0), the forward and backward acoustic power transmission spectra are slightly different particularly around the resonant frequency of the filter as shown in Supplementary Information Fig. S.9. Such difference is originated from the non-ideal acoustic absorption condition at the two ends of our wave-guiding system and the resulted stand-wave fluctuations recorded at the acoustic probe/detector.

As discussed theoretically above, our proposed system can experience a transition from symmetric transmission into unidirectional transmission with the blade’s increasing rotation frequency. When the blade is rotating at a frequency of 15 Hz (Fig. 4(d)), acoustic power transmission spectra for the two directions is nearly identical. Such experimental behavior is different from what is observed in the simulation shown in Fig. 3(b), which is resulted from the broader bandwidth of the Helmholtz filter used in the experiment as discussed above. When the rotation frequency is increased to *f*_*r*_ = 40 *Hz* and *f*_*r*_ = 65 *Hz*, the generated acoustic first order scattering frequency components start to acquire acoustic frequency falling out of the filter’s stop-band, which results in distinct asymmetric transmitted power for the two propagation directions as shown in Fig. 4(c,d). Compared with the ~100 dB power transmission contrast ratio in the simulation at the filter’s center frequency as shown in Fig. 3(d), the contrast ratio for the two propagation directions obtained in the experiment is much smaller than the theoretical predictions due to the relative low scattering conversion efficiency and large propagation loss. Nevertheless, at the blade’s highest rotation frequency of 65 Hz in our experiment, it still can be as large as 20 dB, which is adequate for many practical applications.

## Discussion

We demonstrate an acoustic scattering effect from a time-varying medium (a rotating elliptical blade). Both our theoretical study and experimental investigation suggest that the state of the blade plays a critical role in achieving novel acoustic transmission behavior. When this blade is at rest without any motion, the system is linear and time-independent, which only exhibits symmetric power transmission for two propagation directions as indicated in Figs. 3(a). However, as soon as the blade starts to rotate, acoustic scattering takes place and part of the input acoustic wave is scattered into harmonic frequency components as shown in Fig. 1, which subsequently transmit through the Helmholtz filter in a forward propagation configuration. However, in a backward propagation, for the input acoustic wave with frequency falling into the filter’s stop-band, the filter greatly attenuates the input power leading to almost no power transmission. This asymmetric direction-dependent power transmission phenomenon manifests the acoustic unidirectionality in our system.

In our system design, the use of the time-varying medium is very advantageous, because it allows for great flexibility in manipulating acoustic scattering process and in optimizing the performance of our unidirectional system. The scattering frequency shift is determined by the rotating frequency of the blade, which can be intentionally modified to a certain extent, the frequency of scattering frequency components can also be tuned accordingly. This unique feature in our system implies that the contrast ratio can also be deliberately designed (see Supplementary Information Fig. S.7.). In addition, such kind of time-varying medium can be exploited to construct complicated acoustic structures, e.g., a phase-conjugated time-varying medium array or an acoustic resonator consisting of acoustic time-varying medium, to solve the low conversion efficiency issue in the time-varying acoustic scattering process in our current configuration and consequently to further improve the contrast ratio for the unidirectional transmission.

Since different rotating angles of the elliptical blade correspond to different effective densities (see Supplementary Information Fig. S.1.) and thus different effective phases, the acoustic time-varying medium concept can also be extended to investigate many dynamic phase modulation related physical phenomena. These could include quantum geometric phase caused by non-adiabatic evolution, and gauge magnetic potential^27^,^28^ with Aharonov-Bohm effects in electronic or photonic time-varying systems. Moreover, by utilizing phase conjugated time-varying modulation, nonreciprocal transmission devices such as acoustic isolators^3^,^27^ also might be expected in the future.

In conclusion, we demonstrate a frequency shift effect in a linear time-varying system based on the interaction of an acoustic wave with a rotating elliptical blade. In such a system, an incident acoustic wave is scattered by a rotating blade, and the resulted scattering wave experiences harmonic frequency shift. We show that such frequency conversion process can be described by a method that we developed based on the linear time-varying scattering theory, although there are some discrepancies between theory and experiment, which needs further investigation. Furthermore, by combining such frequency shift effect with efficient acoustic filtering, we realize acoustic unidirectional propagation with 20 dB contrast ratio between two counter propagation directions at audible frequencies, which can be tuned by varying the blade’s rotation frequency. It is worth noting that our concept of using an acoustic time-varying medium can be equally applied to frequency ranges other than audible frequencies. We believe our concept of linear time-varying acoustic scattering and the design of such unidirectional transmission device may find applications in relevant fields such as non-destructive testing of turbulent flow pipe, acoustic imaging, audible signal processing, and etc. Moreover, the underlying mechanism of this time-varying acoustic scattering is linked to acoustic dynamic phase modulation process. In other words, by taking advantage of dynamic phase modulation, novel physical phenomena such as gauge magnetic potential^3^,^9^ in condensed matter might be simulated by a carefully designed macroscopic acoustic time-varying system.

## Methods

### Full-wave simulations

The full-wave numerical simulations of the acoustic wave transmission were performed using the aeroacoustics module of the commercially available finite-element software COMSOL Multiphysics. The rotating blade was modeled as a mathematically imposed and time-varying area inside the waveguide. Rigid wall boundary conditions were used for the waveguide except for the two ends, which were terminated with plane-wave scattering boundary conditions. For acoustic excitation, a plane-wave pressure field was used at the left end boundary of the waveguide. The time varying acoustic scattering process of the blade was studied in time domain with a transient solver in COMSOL using a time step 10^−6^ s. Resonance frequency of the Helmholtz resonator array was determined with a steady state solver. Transmission spectra were obtained by performing a set of frequency domain simulations with a frequency increment of 1 Hz.

### Acoustic measurement methods

Our experimental investigation was carried out in an acoustic waveguide. In order to reduce the reflection at the end of the waveguide caused by impedance mismatch, the end was sealed with sound absorption sponges. The measured absorption efficiency of the absorption layer is provided in Supplementary Information. We used an acoustic pressure probe (B&K-4939-2670 microphone) and a signal analyzer (B&K-3560-C) for acoustic signal acquisition and frequency analysis. More details for measurements can be found in Supplementary Information.

## Additional Information

**How to cite this article**: Wang, Q. *et al*. Acoustic Asymmetric Transmission Based on Time-Dependent Dynamical Scattering. *Sci. Rep*. **5**, 10880; doi: 10.1038/srep10880 (2015).

## Supplementary Material

Supplementary Information

Supplementary Video
