# Supplementary material for: Acoustic asymmetric transmission based on time-dependent dynamical scattering
Source: Sci Rep. 2015 Jun 3;5:10880. doi: 10.1038/srep10880 (PMC4454088; doi:10.1038/srep10880)
Supplement: Supplementary Information [file srep10880-s1.pdf]

Supplementary Information for Acoustic Asymmetric Transmission Based on Time-Dependent Dynamical Scattering

Qing Wang[1]*, Yang Yang[1], Xu Ni[1], Ye-Long Xu[1], Xiao-Chen Sun[1], Ze-Guo Chen[1], Liang Feng[2], Xiao-ping Liu[1], Ming-Hui Lu[1], Yan-Feng Chen[1]

1. *National Laboratory of Solid State Microstructures & Department of Materials Science and Engineering, Nanjing University, Nanjing 210093, China*
2. *Department of Electrical Engineering, The State University of New York at Buffalo, Buffalo, NY 14260, USA*

Theory

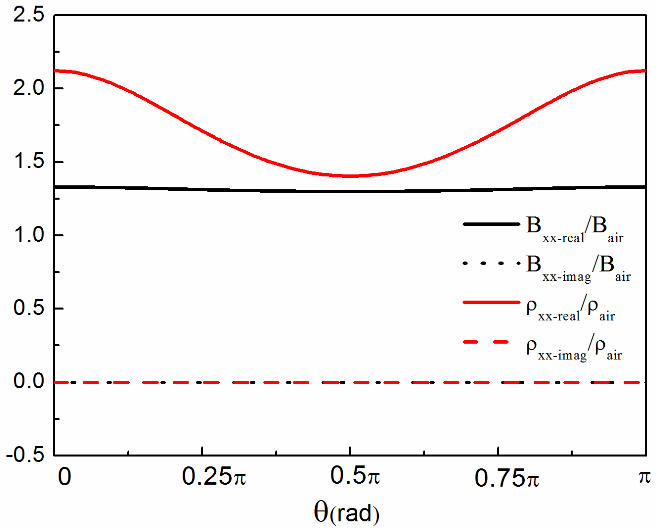

S.1. Rotation-angle dependent effective material parameters of the rotating elliptical blade region obtained via Full-wave simulation. The time-varying medium in our system is anisotropic and thus tensors should be used in perturbation theory. $B_{xx-real}$ ($\rho_{xx-real}$) and $B_{xx-imag}$ ($\rho_{xx-imag}$) correspond to real and imaginary part of effective modulus (effective density) tensor element responsible for the collinear scattering process along x-axis studied in this work. B_{air} and ρ_{air} are the modulus and density of air respectively.

Measuring the effective parameters of the time-varying medium is significant in interpreting our results. After a more in-depth consideration of this kind of measurement, we discovered several technical difficulties, which prevent us from obtaining the experiment proof.

1) The acoustic field of the effective region might be inhomogeneous, and thus just directly measuring field at several locations in the effective region cannot reflect the whole scenario.

2) With the limits of present measurement techniques available, it is nearly impossible to directly measure the whole sound field of the time-varying region as a function of time. Neither laser interferometer nor laser Doppler can directly measure the dynamic parameters of this optical reflection-less air medium. Schlieren device has little use here because the acoustic field in our experiment is too weak to be measured. Because the phase distribution is not a stable one, detector array also cannot work in such a dynamical time-varying case for imaging the acoustic field.
3) Transient transmission and reflection method for deducing parameters has its own problem here. In experiment, recording time of signal is finite. Take a sinusoidal signal with a length of ΔT as an example (Eq. (1)), which resembles the case in our experiment.

$$p=\begin{cases} 0, & t<-\Delta T/2 \\ p_0\cos(\omega_0 t), & -\Delta T/2\le t\le \Delta T/2 \\ 0, & t<\Delta T/2 \end{cases}; \quad (1)$$

The amplitude of wave is obtained by Fourier Transform. The frequency component of signal, however, is no longer a single frequency. The frequency spectrum will have a certain bandwidth of $\Delta\omega$. $\Delta\omega$ should satisfy the equation:

$$\Delta\omega \bullet \Delta T = 2\pi, \quad (2)$$

which can also be expressed as

$$\Delta f \bullet \Delta T = 1. \quad (3)$$

If the recording time of signal is too short (to deduce the time-varying parameters, time axis of transient signals should be cut to the small enough parts to match the modulation frequency), we could not get the exact amplitude and phase of pressure in the effective rotation region, which means it is impossible to use this method deducing the effective time-varying parameters.

Taking all the above factors into consideration, we resort to the quasi-static measurement for the effective parameters as shown in Fig.S.3. Since air turbulence in the waveguide caused by the elliptic shape of the blade is minimal, slowly varying approximation with quasi-static effective parameters can still be used in our analysis. Our transmission experimental results in fact well support our theoretical findings.

Since the reviewer still has concerns about our quasi-static method, we feel it is necessary for us to elaborate it in more details and hope this will clarify some of the issues that the reviewer may have in his/her mind. The effective parameters [*Phys. Rev. B.* **76**, 144302 (2007)] of the time-varying region in our work, such as density and acoustic velocity, are deduced from acoustic transmission and reflection fields by utilizing the continuity equation. In theory, as $p = Z g v$ where p is sound pressure, Z is acoustic impedance of medium and v is vibration velocity, both sound pressure and vibration velocity can be used to deduce effective parameters. Nevertheless, the vibration velocity of a medium, *e.g.*, air, subjected to an acoustic field is too difficult to be measured directly. Laser interferometer or laser Doppler vibration meter may be used if there is

measurable optical reflection, which is not the case here for air. An alternative way used by many scientists is to measure sound pressure directly with microphones for fluid media like air. Once the phase and amplitude of the pressure fields before and after the time-varying medium (shown in Fig.S.2) are known. The effective parameters of the time-varying medium can be derived. Detailed steps are discussed as follows.

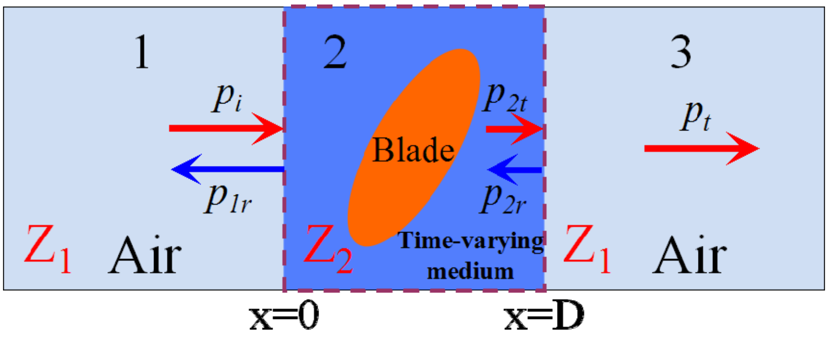

S.2. Schematic of a waveguide section with three regions. Medium in region 1 and region 3 is air while region 2 contains the time-varying medium. Here, the length D of the effective medium region is 0.02m in both simulation and experiment. The blade is placed in the center of this region.

A three-region waveguide structure under study is shown in Fig.S.2. Medium in region 1 and region 3 is static air while region 2 contains the time-varying medium. We will adopt the continuity equation to deduce the effective mass density of this time-varying medium.

Step One: We need to figure out the expressions for the pressure and velocity in every region.
Expressions of region 1:

In region 1, sound pressure of incident acoustic waves (p_i) and air's vibration velocity of incident acoustic waves (v_i) can be written as:

$$\begin{cases} p_i = p_{ia} e^{j(\omega t - k_1 x)} \\ v_i = v_{ia} e^{j(\omega t - k_1 x)} \end{cases}, \text{ where } k_1 = \omega / c_1 \; ; \qquad (4)$$

In region 1, sound pressure of reflected acoustic waves (p_{1r}) and air's vibration velocity of reflected acoustic waves (v_{1r}) can be written as:

$$\begin{cases} p_{1r} = p_{1ra} e^{j(\omega t + k_1 x)} \\ v_{1r} = v_{1ra} e^{j(\omega t + k_1 x)} \end{cases}, \text{ where } k_1 = \omega / c_1 \; ; \qquad (5)$$

Expressions of region 2:

In region 2, sound pressure of transmission acoustic waves (p_{2t}) and air's vibration velocity of transmission acoustic waves (v_{2t}) can be written as:

$$\begin{cases} p_{2t} = p_{2ta} e^{j(\omega t - k_2 x)} \\ v_{2t} = v_{2ta} e^{j(\omega t - k_2 x)} \end{cases}, \text{ where } k_2 = \omega / c_2 ; \tag{6}$$

In region 2, sound pressure of reflected acoustic waves (p_{2t}) and air's vibration velocity of reflected acoustic waves (v_{2t}) can be written as:

$$\begin{cases} p_{2r} = p_{2ra} e^{j(\omega t + k_2 x)} \\ v_{2r} = v_{2ra} e^{j(\omega t + k_2 x)} \end{cases}, \text{ where } k_2 = \omega / c_2 ; \tag{7}$$

Expressions of region 3:

In region 3, sound pressure of transmission acoustic waves (p_t) and air's vibration velocity of transmission acoustic waves (v_t) can be written as:

$$\begin{cases} p_t = p_{ta} e^{j[\omega t - k_1 (x - D)]} \\ v_t = v_{ta} e^{j[\omega t - k_1 (x - D)]} \end{cases}, \text{ where } k_1 = \omega / c_1 . \tag{8}$$

Step Two: By applying boundary conditions at boundary $x = 0$, we get the following two equations,

$$\begin{cases} p_{ia} + p_{1ra} = p_{2ta} + p_{2ra} \\ v_{ia} + v_{1ra} = v_{2ta} + v_{2ra} \end{cases} ; \tag{9}$$

Similarly at the boundary $x = D$, we get

$$\begin{cases} p_{2ta} e^{-jk_2 D} + p_{2ra} e^{jk_2 D} = p_{ta} \\ v_{2ta} e^{-jk_2 D} + v_{2ra} e^{jk_2 D} = v_{ta} \end{cases} . \tag{10}$$

Acoustic pressure, acoustic impedance of medium and vibration velocity of medium are related according to,

$$\begin{cases} v_{ia} = p_{ia} / Z_1 \\ v_{1ra} = -p_{1ra} / Z_1 \end{cases} , \tag{11}$$

where $Z_1 = \rho_1 c_1$ is the acoustic impedance of medium in region 1;

$$\begin{cases} v_{2ta} = p_{2ta} / Z_2 \\ v_{2ta} = -p_{2ta} / Z_2 \end{cases} , \tag{12}$$

where $Z_2 = \rho_2 c_2$ is the acoustic impedance of medium in region 2;

$$v_{ta} = p_{ta} / Z_1 , \tag{13}$$

where $Z_1 = \rho_1 c_1$ is the acoustic impedance of medium in region 3;

By substituting Eq. (11), (12) and (13) into Eq. (9) and Eq. (10), we get:

$$\begin{cases} p_{2ta} = \frac{1}{2}[p_{ia} + p_{1ra} + \frac{Z_2}{Z_1}(p_{ia} - p_{1ra})] \\ p_{2ra} = \frac{1}{2}[p_{ia} + p_{1ra} - \frac{Z_2}{Z_1}(p_{ia} - p_{1ra})] \end{cases}; \quad (14)$$

$$\begin{cases} p_{2ta} = \frac{e^{jk_2 D}}{2}[p_{ta} + \frac{Z_2}{Z_1} p_{ta}] \\ p_{2ra} = \frac{e^{-jk_2 D}}{2}[p_{ta} - \frac{Z_2}{Z_1} p_{ta}] \end{cases}. \quad (15)$$

By substituting Eq. (14) into Eq. (15) and eliminating p_{2ta} and p_{2ra}, we get the following equations

$$\begin{cases} (Z_1 + Z_2) p_{ia} + (Z_1 - Z_2) p_{1ra} = e^{jk_2 D}(Z_1 + Z_2) p_{ta} \\ (Z_1 - Z_2) p_{ia} + (Z_1 + Z_2) p_{1ra} = e^{-jk_2 D}(Z_1 - Z_2) p_{ta} \end{cases}; \quad (16)$$

By solving Eq. (16), we can express Z_2 in terms of $Z_1, p_{ia}, p_{1ra}, p_{ta}$ as follows

$$Z_2 = \left[\frac{(p_{ia} + p_{1ra})^2 - p_{ta}^2}{(p_{ia} - p_{1ra})^2 - p_{ta}^2} \right]^{\frac{1}{2}} Z_1. \quad (17)$$

Step Three: By substituting Eq. (17) into Eq. (16) and eliminating Z_2, we will obtain the detailed description of k_2. Thus, we calculate the effective parameters ρ_2 and B_2 by substituting the results of k_2 and Eq. (17) into the equations as follows,

$$\begin{cases} c_2 = \omega / k_2 \\ B_2 = Z_2 c_2 \\ \rho_2 = Z_2 / c_2 \end{cases}, \quad (18)$$

where ω is the angular frequency of incident waves.

It is easy to extract the pressures of incident, transmitted and reflected pressure fields to retrieve the effective parameters in simulation (see Fig. S.1). However, it is a little complicated to measure directly the exact values of incident and reflected pressures in experiment, because incident waves and reflected waves are always mixed up together ($p_1 = p_i + p_{1r}$). In this case, Eq. (17) can be changed to:

$$Z_2 = \left[\frac{(1+r)^2 - t^2}{(1-r)^2 - t^2} \right]^{\frac{1}{2}} Z_1, \quad (19)$$

Where $r = {p_{1ra}}/{p_{ia}}$ and $t = {p_{ta}}/{p_{ia}}$.

The reflected coefficient (r) and transmission coefficient (t) have to be measured with the Transfer-function Method [ISO,10534-2-1998:Acoustics Determination of Sound Absorption Coefficient and Impedance in Impedance Tubes. Part 2: Transfer-function Method (ISO, Geneva, Switzerland, 1998)].

By repeating the same procedure at the blade's different rotation angle, we can deduce the angle-dependent effective parameters as shown in Fig. S.3. Despite the absolute value for measured and simulated effective modulus and density are slightly different due to the loss in the experiments, harmonically varying effective parameters as a function of the rotation angle is clearly seen in both experiment and simulation.

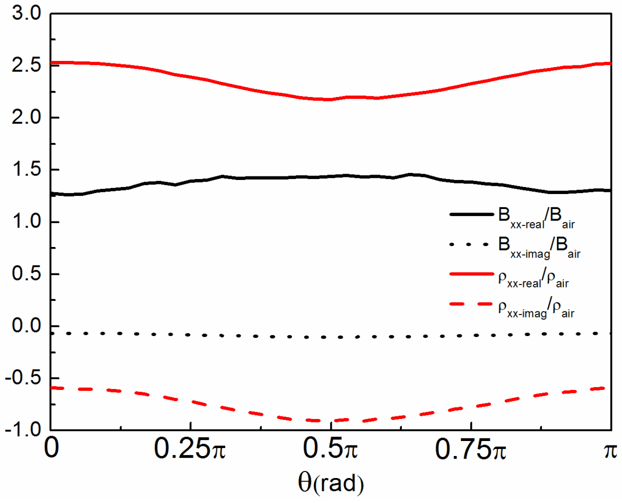

S.3. Experimentally deduced effective parameters. The time-varying medium in our system is anisotropic and thus tensors should be used in perturbation theory. $B_{xx-real}$ ($\rho_{xx-real}$) and $B_{xx-imag}$ ($\rho_{xx-imag}$) correspond to real and imaginary part of effective modulus (effective density) tensor element responsible for the collinear scattering process along x-axis studied in this work. B_{air} and ρ_{air} are the modulus and density of air respectively.

To conclude, the deduced effective parameters for the time-varying medium are consistent with the simulation to great extent, and their periodic change in time as a result of rotation is responsible for the phenomenon of dynamic modulation or dynamic scattering observed in our work. We would love to measure the effective parameters directly. The current available technology, however, prevents us from doing that. In contrast, the quasi-static model we adopted in this work works well, and the simulation results obtained from such model matches with the experimental transmission spectrum to large extent.

In the presence of rotation, the linear acoustic equations can be written as

$$\frac{\partial \rho}{\partial t}+\rho\nabla\cdot\vec{v}=0 \tag{20}$$

$$\rho\frac{\partial \vec{v}}{\partial t}+\nabla p=0 \tag{21}$$

$$\frac{\partial p}{\partial t}=\sum_{i=xx,xy,yx,yy} c_i^2\frac{\partial \rho}{\partial t} \tag{22}$$

.

Here equations (20), (21) and (22) represent equation of mass conservation, equation of momentum conservation and thermodynamic equation of state, respectively. ρ is the time dependent density of the medium and c_0 is the speed of sound, which can be treated as an invariant. $\vec{v}$ is the velocity field perturbation of the acoustic wave and p is the acoustic pressure. From Eqs. (20-22) we get

$$\frac{\partial^2 p}{\partial^2 t}-\frac{\partial(\rho c_0^2)}{\partial t}\cdot\frac{1}{\rho c_0^2}\frac{\partial p}{\partial t}-\rho c_0^2\nabla\left(\frac{1}{\rho}\nabla p\right)=0\,, \tag{23}$$

Where $\rho=\begin{bmatrix}\rho_{xx} & \rho_{xy}\\ \rho_{yx} & \rho_{yy}\end{bmatrix}=\begin{bmatrix}\rho_{xx0}+\rho_{xx}(t) & \rho_{xy0}+\rho_{xy}(t)\\ \rho_{yx0}+\rho_{yx}(t) & \rho_{yy0}+\rho_{yy}(t)\end{bmatrix}=\rho_0+\rho_1(t)$,here ρ_0 and $\rho_1(t)$ are tensors..

By substituting Eq. (22) into Eqs. (20) and (21), and replacing velocity field $\vec{v}$ by velocity potential $\vec{v}=\nabla\phi$, the equations can be described as

$$\left(H_0+H_1(t)\right)|\psi\rangle=\omega|\psi\rangle \tag{24}$$

where ω is the eigenfrequency, and H_0 and $H_1(t)$ can be expressed as

$$H_0=\begin{bmatrix}0 & c_0^2\rho_0\partial_x & c_0^2\rho_0\partial_y\\ H_{210} & 0 & 0\\ H_{310} & 0 & 0\end{bmatrix}$$

$$H_1(t)=\begin{bmatrix}0 & c_0^2\rho_1(t)\partial_x & c_0^2\rho_1(t)\partial_y\\ H_{21}(t) & 0 & 0\\ H_{31}(t) & 0 & 0\end{bmatrix} \tag{25}$$

$$|\psi\rangle=\begin{pmatrix}p\\ iv_x\\ iv_y\end{pmatrix}.$$

Where

$$\begin{aligned}H_{210}&=\left(\rho_0^{-1}\right)_{xx}\partial_x+\left(\rho_0^{-1}\right)_{xy}\partial_y\\ H_{310}&=\left(\rho_0^{-1}\right)_{yx}\partial_x+\left(\rho_0^{-1}\right)_{yy}\partial_y\end{aligned} \tag{26}$$

$$\begin{aligned}H_{21}(t)&=\left(\rho_1^{-1}(t)\right)_{xx}\partial_x+\left(\rho_1^{-1}(t)\right)_{xy}\partial_y=\left[\left(\rho_1^{-1}\right)_{xx}\partial_x+\left(\rho_1^{-1}\right)_{xy}\partial_y\right]\cos(\omega t)\\ H_{31}(t)&=\left(\rho_1^{-1}(t)\right)_{yx}\partial_x+\left(\rho_1^{-1}(t)\right)_{yy}\partial_y=\left[\left(\rho_1^{-1}\right)_{yx}\partial_x+\left(\rho_1^{-1}\right)_{yy}\partial_y\right]\cos(\omega t)\end{aligned} \tag{27}$$

$$c_0^2\rho=\sum_{i=xx,xy,yx,yy}c_i^2\rho_i=\sum_{i=xx,xy,yx,yy}c_i^2\left(\rho_{i0}+\rho_{i1}(t)\right)=c_0^2\rho_0+c_0^2\rho_1(t) \tag{28}$$

$$\begin{aligned}\rho^{-1}&=\frac{\rho^*}{|\rho|}\approx\frac{\rho_0^*+\rho^*(t)}{|\rho_0|}=\rho_0^{-1}+\rho_1^{-1}(t)=\rho_0^{-1}+\rho_1^{-1}\cos(\omega t)\\ &=\begin{bmatrix}\left(\rho_0^{-1}\right)_{xx} & \left(\rho_0^{-1}\right)_{xy}\\ \left(\rho_0^{-1}\right)_{yx} & \left(\rho_0^{-1}\right)_{yy}\end{bmatrix}+\begin{bmatrix}\left[\rho_1^{-1}(t)\right]_{xx} & \left[\rho_1^{-1}(t)\right]_{xy}\\ \left[\rho_1^{-1}(t)\right]_{yx} & \left[\rho_1^{-1}(t)\right]_{yy}\end{bmatrix},\end{aligned} \tag{29}$$

where ρ^* is the cofactor of ρ.

Considering that effective mass density is dependent on the rotation angle with the approximate cosine profile, so $H_1(t)$ can be expressed as:

$$H_1(t)=\begin{bmatrix}0 & c_0^2\rho_{10}\partial_x & c_0^2\rho_{10}\partial_y\\ \left(\rho_1^{-1}\right)_{xx}\partial_x+\left(\rho_1^{-1}\right)_{xy}\partial_y & 0 & 0\\ \left(\rho_1^{-1}\right)_{yx}\partial_x+\left(\rho_1^{-1}\right)_{yy}\partial_y & 0 & 0\end{bmatrix}\cos(\omega t).$$

In the above analysis, we have set $\rho=\rho_0+\rho_1(t)$, here ρ_0 and $\rho_1(t)$ are tensors.

In the absence of rotation, $H_1(t)=0$ and the eigen equation can be written as

$$H_0 |\psi_n\rangle = \omega_n |\psi_n\rangle_. \tag{30}$$

We can get $|\psi_n\rangle$ easily by solving the corresponding Helmholtz equation.

In the presence of rotation, considering the two-folded symmetry of elliptical blades, the effective Hamiltonian $H_1(t)$ can be written as $H_1 = V\cos(2\omega_r t)$, where ω_r is the rotation angular frequency of the elliptical blade. The new eigenvectors can be written as a linear superposition of $|\psi_n\rangle$:

$$\left(H_0 + V\cos(2\omega_r t)\right)|\psi\rangle = \omega|\psi\rangle \tag{31}$$

$$|\psi\rangle = \sum_n a_n |\psi_n\rangle. \tag{32}$$

The coefficient a_n must satisfy

$$a_n(t) = \frac{1}{i\hbar}\sum_m \int_0^t \langle\psi_n|H_1(t')|\psi_m\rangle e^{i(\omega_n-\omega_m)t'}dt'. \tag{33}$$

When single frequency acoustic wave enters the system, the initial condition satisfies $a_n(0) = \delta_{n0}$. Based on time-dependent perturbation theory and Fermi golden rule, transition rate $w_n = \frac{d|a_n|^2}{dt}$ can be expressed as

$$w_n = 2\pi\left|\langle\psi_n|V|\psi_0\rangle\right|^2 \delta(\omega_n - \omega_0 \pm 2\omega_r) \tag{34}$$

The above equation means that the initial system energy level will be split to create two additional sub energy levels, of which the angular frequency are determined according to the following expression,

$$\omega_\pm = \omega_0 \pm 2\omega_r. \tag{35}$$

<u>Details for Experiments</u>

As shown in Figure.S.4, the acoustic waveguide is made of aluminum alloy. Its length is $1.5m$ and its rectangular cross-section has an outside dimension $25mm \times 100mm$. The thickness of the wall is $1.25mm$. The waveguide is filled with air at room temperature. The normal frequency

of this waveguide can be calculated by $f_{n_x n_y} = \frac{c_0}{2}\sqrt{\left(\frac{n_x}{l_x}\right)^2 + \left(\frac{n_y}{l_y}\right)^2}$, where $l_x = 22.5mm$ and $l_y = 97.5mm$ are the inner width and height of the acoustic waveguide. The single-mode cut-off frequency of this waveguide can then be determined to be $f_{(1,0)} = 7622Hz$. Therefore as long as the input frequency $f < f_{(1,0)}$, the waveguide can support single mode operation. The meta-atom is an elliptical blade made of nylon with a density of 1150kg/m^3 and an acoustic velocity of 2600m/s, which is attached to the rotation axis of a DC motor. A modulator controls the rotation frequency of the motor with a frequency resolution of 1Hz. The distance between the meta-atom (blade) and the Helmholtz resonator array (measured from blade's rotation axis to the central axis of the nearest resonator) is 450mm. The Helmholtz resonator array has a pitch about 50 mm. Each individual Helmholtz resonator is made of ABS plastics with a density of 1050kg/m^3 and an acoustic velocity of 2450m/s via 3D printing. The end of the acoustic waveguide was sealed with a 145-mm-thick sponge. Its total absorption along with several other tested seal configurations is shown in Fig. S.4. A probe (B&K-4939-2670 microphone) is placed inside the waveguide via a drilled hole on the waveguide's ceiling for acoustic signal acquisition. Collected signal data are processed by B&K-3560-C signal analyzer.

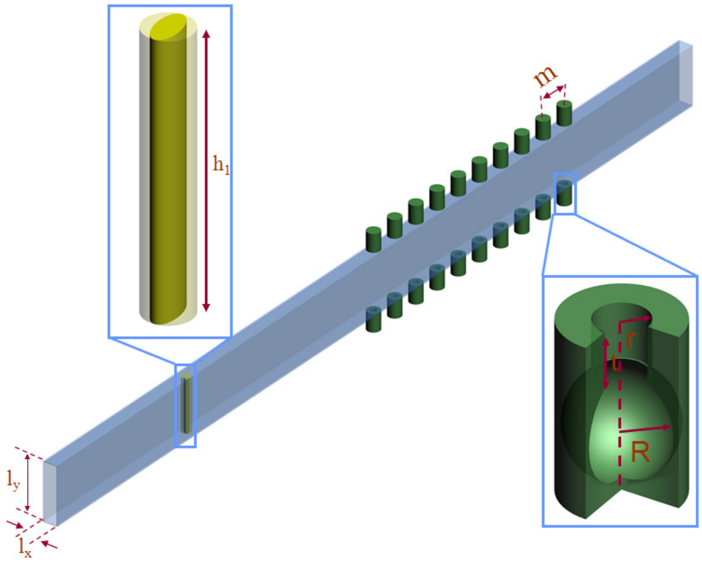

S.4. Geometric parameters of the unidirectional system.

$h_1 = 90mm; l_x = 22.5mm; l_y = 97.5mm; m = 50mm; R = 10mm; r = 5mm; t = 10mm$.

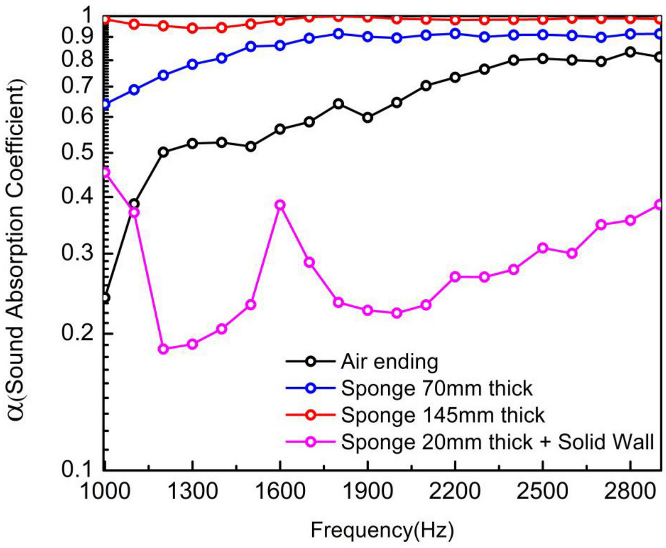

S.5. Acoustic absorption coefficient for various termination conditions. Since 145-mm-thick sponge has the best absorption performance among these four termination conditions, it was used exclusively in our experimental investigation to seal the waveguide.

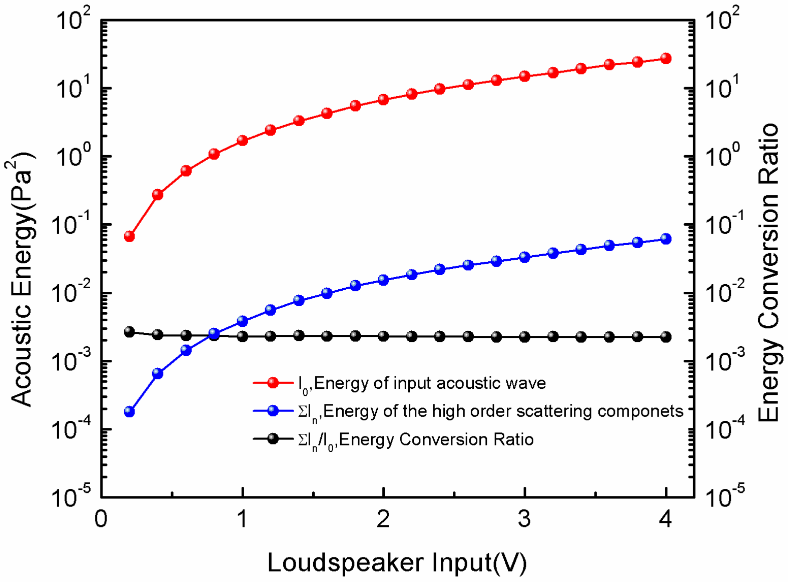

S.6. Experimental results for acoustic transmission and energy conversion ratio through the elliptical blade as a function of loudspeaker input voltage. I_0 is the input energy of the input acoustic wave and $\sum I_n$ are the sum of the output energy of all the high order scattering components generated via time-varying acoustic scattering. Energy conversion ratio here is defines as $\sum I_n / I_0$. From the data shown in this figure, the time-varying acoustic scattering we

have mentioned is a kind of linear effects.

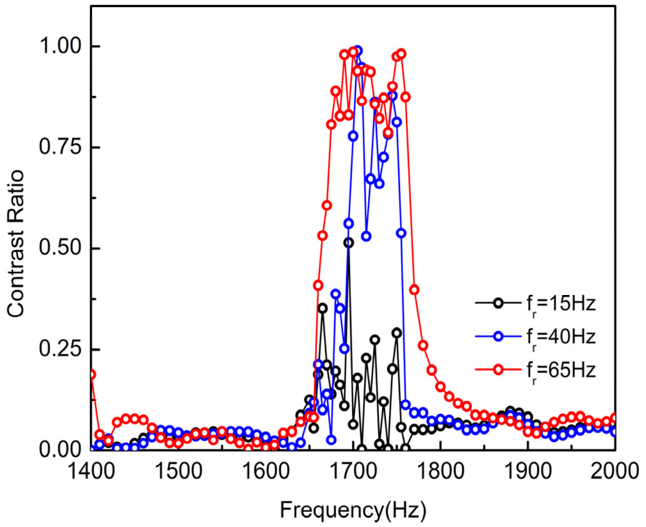

S.7. Contrast ratio for three different rotation frequencies $f_r = 15Hz, 40Hz, 65Hz$**.** Here the contrast ratio is defined as $R_c = \frac{|I_{forward} - I_{backward}|}{I_{forward} + I_{backward}}$, where $I_{forward}$ and $I_{backward}$ are the integrated output energy for two forward and backward propagation directions.

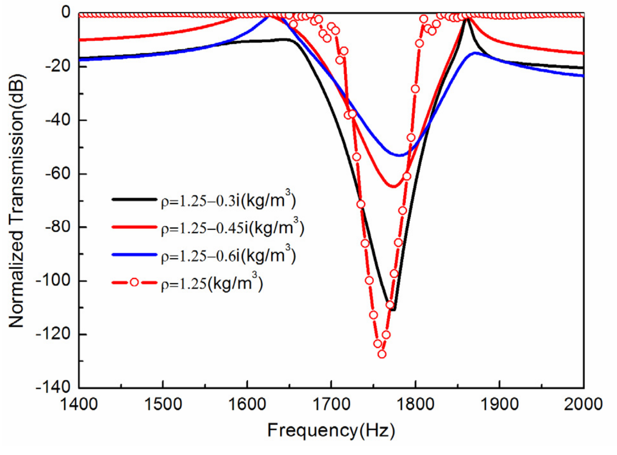

S.8. Normalized transmission curves for the band-stop filters considering air loss via full wave simulations. Air loss can not be ignored in experiments. We defined the mass density in resonance cavity as $1.25kg/m^3$, $(1.25-0.3i)kg/m^3$, $(1.25-0.45i)kg/m^3$ and $(1.25-0.6i)kg/m^3$, respectively. The results can give an explanation for the differences in filters' performance between simulations and experiments.

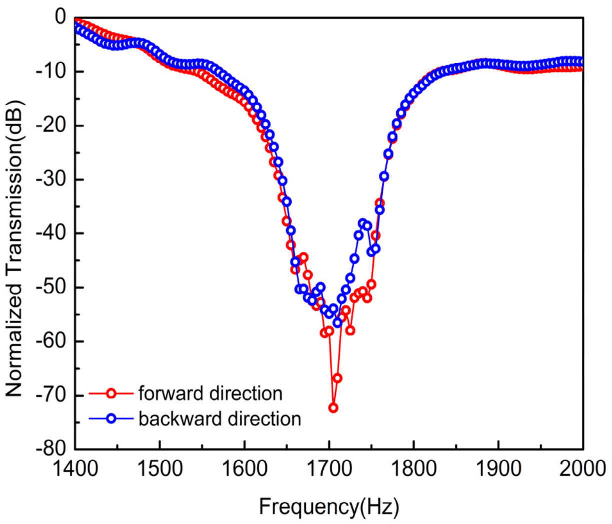

S.9. Normalized transmission for the case, where the blade is placed in the acoustic waveguide but is at rest with $f_r = 0$. As the absorption coefficient at the end of the acoustic waveguide is not 100 percents, transmission spectra from two directions (forward and backward) are not the same in the case the meta-atom is placed in the waveguide but is at rest with $f_r = 0$. In this case, we can calculate correction factors as a function of frequency by doing division to adjust results to ideal conditions. When the blade is driven by the motor, the transmission spectra need to be normalized by the correction factor function got from the case when $f_r = 0$.
