# Supplementary figures and images for: Acoustic asymmetric transmission based on time-dependent dynamical scattering
Source: Sci Rep. 2015 Jun 3;5:10880. doi: 10.1038/srep10880 (PMC4454088; doi:10.1038/srep10880)

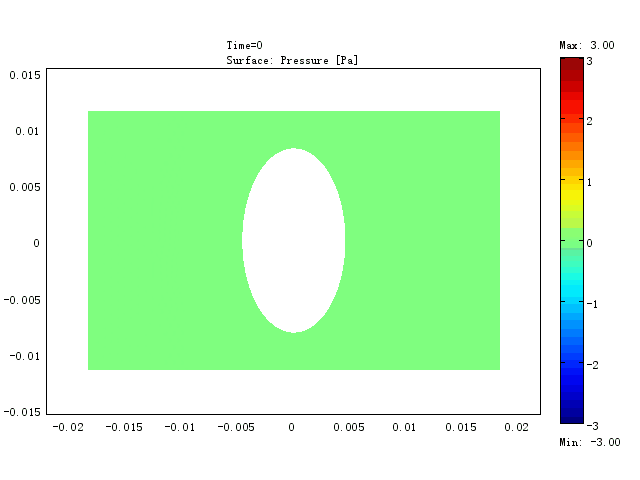

Supplement: Supplementary Video [file srep10880-s2.gif]
